# Effect of age and diet composition on activity of pancreatic enzymes in birds

**DOI:** 10.1007/s00360-012-0731-2

**Published:** 2012-12-27

**Authors:** Paweł Brzęk, M. Eugenia Ciminari, Kevin D. Kohl, Krista Lessner, William H. Karasov, Enrique Caviedes-Vidal

**Affiliations:** 1 Department of Forest and Wildlife Ecology, University of Wisconsin, 1630 Linden Drive, Madison, WI 53706 USA; 2 Laboratorio de Biología “Professor E. Caviedes Codelia”, Facultad de Ciencias Humanas, Universidad Nacional de San Luis, 5700 San Luis, Argentina; 3 Departamento de Bioquímica y Ciencias Biológicas, Facultad de Química, Bioquímica y Farmacia, Universidad Nacional de San Luis, 5700 San Luis, Argentina; 4 Instituto Multidisciplinario de Investigaciones Biológicas de San Luis, Consejo Nacional de Investigaciones Científicas y Técnicas, 5700 San Luis, Argentina; 5 Institute of Biology, University of Białystok, Świerkowa 20B, 15-950 Białystok, Poland; 6 Department of Biology, University of Utah, 257 South 1400 East, Salt Lake City, UT 84112 USA

**Keywords:** Digestive enzymes, Pancreas, Ontogeny, Developmental flexibility, Diet composition

## Abstract

Digestive enzymes produced by the pancreas and intestinal epithelium cooperate closely during food hydrolysis. Therefore, activities of pancreatic and intestinal enzymes processing the same substrate can be hypothesized to change together in unison, as well as to be adjusted to the concentration of their substrate in the diet. However, our knowledge of ontogenetic and diet-related changes in the digestive enzymes of birds is limited mainly to intestinal enzymes; it is largely unknown whether they are accompanied by changes in activities of enzymes produced by the pancreas. Here, we analyzed age- and diet-related changes in activities of pancreatic enzymes in five passerine and galloanserine species, and compared them with simultaneous changes in activities of intestinal enzymes. Mass-specific activity of pancreatic amylase increased with age in young house sparrows but not in zebra finches, in agreement with changes in typical dietary starch content and activity of intestinal maltase. However, we found little evidence for the presence of adaptive, diet-related modulation of pancreatic enzymes in both passerine and galloanserine species, even though in several cases the same birds adaptively modulated activities of their intestinal enzymes. In general, diet-related changes in mass-specific activities of pancreatic and intestinal enzymes were not correlated. We conclude that activity of pancreatic enzymes in birds is under strong genetic control, which enables evolutionary adjustment to typical diet composition but is less adept for short term, diet-related flexibility.

## Introduction

Effective function of the gastrointestinal tract is important in birds due to the high energetic demands set by their flight ability and fast growth rate (Karasov and Wright 2002; Caviedes-Vidal et al. 2007; McWhorter et al. 2009). The key steps in food hydrolysis are performed by digestive enzymes secreted by the pancreas and others located on the intestinal brush-border epithelium, with products of pancreatic enzymes often being the substrates for intestinal enzymes (Karasov and Hume 1997). Typically, food is first hydrolyzed by pancreatic enzymes that break starch to disaccharides [via amylase (EC 3.2.1.1)], and proteins to peptides [via trypsin (EC 3.4.21.4) and chymotrypsin (EC 3.4.21.1)]. Subsequently, intestinal brush-border enzymes hydrolyze disaccharides into monosaccharides [maltase (EC 3.2.1.20) and sucrase (EC 3.2.1.48)], and peptides into amino acids [e.g., aminopeptidase-N; APN (EC 3.4.11.2)]. Two important evolutionary hypotheses can be proposed based on the described course of the digestion process: (1) the activity of digestive enzymes should be adjusted to the content of their substrates in food, such that organisms do not waste energy on enzymes in excess of need, but at the same time fully utilize available resources (‘adaptive modulation hypothesis’; Karasov and Diamond 1988); (2) for the same reason, activities of pancreatic and intestinal enzymes processing the same substrate should be tuned to each other, a prediction analogous to the more general idea of symmorphosis (sensu Weibel 2000).

In agreement with the first prediction, many studies have found significant correlation between activities of digestive enzymes and the dietary content of their substrates at both the inter- and intra-specific level (reviewed in Karasov and Hume 1997; Karasov et al. 2011). In passerine birds, there is an inter-specific correlation between diet composition and activity of intestinal and pancreatic carbohydrases, but not proteases (Kohl et al. 2011; Ramirez-Otarola et al. 2011). Within species, adult passerines seem able to adjust activity of intestinal proteases to diet composition, but not intestinal carbohydrases, whereas members of Galloanserae (the clade including galliforms and anseriforms) seem able to modulate activity of their carbohydrases but usually do not modulate proteases (reviewed in McWhorter et al. 2009). Flexibility of digestive enzymes can also be related to feeding ecology and age. For example, young house sparrows (*Passer domesticus*) undergo a gradual switch from an insect-based, carbohydrate-poor diet to a diet containing more seeds (and thus carbohydrate-rich; Anderson 2006). Activity of maltase in house sparrows increases significantly during the nestling period, and is higher when starch is present in the diet (Brzęk et al. 2009, 2011). On the other hand, zebra finches (*Taeniopygia guttata*) consume only seeds after hatching and show much lower ontogenetic changes in the activity of maltase, which are also not affected by higher dietary starch content (Brzęk et al. 2010). However, neither adult house sparrows (Caviedes-Vidal et al. 2000), nor adult zebra finches (Brzęk et al. 2010) show dietary adaptive modulation of maltase activity.

As we explained earlier, diet-related or ontogenetic changes in activities of intestinal enzymes can be hypothesized to be accompanied by parallel changes in the activities of enzymes produced by the pancreas. Indeed, mass-specific activities of both pancreatic and intestinal carbohydrases increase during ontogeny in young house sparrows (Caviedes-Vidal and Karasov 2001), and are significantly correlated to dietary starch content on an inter-specific level in adult passerines (Kohl et al. 2011). However, little is known about the flexibility of pancreatic enzymes in altricial birds. We are aware of only two studies in parulid warblers that produced contradicting results: one of them reported (Levey et al. 1999), but another failed to find (Ciminari et al. 2001) an adaptive flexibility of pancreatic enzymes. Such modulation is also absent in altricial, feral pigeons (Ciminari et al. 2005). However, in precocial poultry species some pancreatic enzymes can be modified adaptively by diet composition (Imondi and Bird 1967; Hulan and Bird 1972; Maiorka et al. 2004).

In the present paper, we describe results of eight trials in which we tested the effects of diet composition and age on the activities of three main pancreatic enzymes: amylase, trypsin, and chymotrypsin, in five species of birds belonging to either altricial passerines or precocial Galloanserae. Five trials were carried out on nestling and adult house sparrows and zebra finches that were subjects of our earlier experiments on intestinal enzymes (Caviedes-Vidal et al. 2000; Brzęk et al. 2009, 2010, 2011). In these trials, we expected that changes in the activities of pancreatic enzymes should parallel those observed for intestinal enzymes, and thus depend on species feeding ecology and age of studied birds. More specifically, we predicted that: (i) activity of amylase should increase with age in house sparrow but not in zebra finch nestlings; (ii) activity of amylase should be higher in house sparrow nestlings fed on starch-containing diet than in birds fed on starch-free diet, whereas diet composition should exert little or no effect on the activity of amylase in adult house sparrows and both nestling and adult zebra finches; (iii) activities of pancreatic trypsin and chymotrypsin should be modulated in parallel with the various patterns for intestinal APN observed in previous studies: no changes in young house sparrows and zebra finches (Brzęk et al. 2009, 2010), but significant and parallel changes with the addition of dietary protein in adults of both species (Caviedes-Vidal et al. 2000; Brzęk et al. 2010). In the last three trials, we analyzed dietary flexibility of pancreatic enzymes in young chickens (*Gallus gallus*), common quails (*Coturnix coturnix*), and mallards (*Anas platyrhynchos*) fed on diets similar to those offered to house sparrows and zebra finches. Here, we could check whether the difference between passerines and the Galloanserae in diet-related flexibility of intestinal carbohydrases and proteases is paralleled by a similar difference in flexibility of pancreatic enzymes.

## Methods

Pancreatic samples analyzed in the present paper came from birds that were subjects of several trials carried out at the University of Wisconsin, Madison, and the University of San Luis, Argentina, some of which were already described in detail elsewhere (see below). In general, birds in all trials were maintained under laboratory conditions and fed on semi-synthetic diets of different composition. These diets differed mainly in the percentage of carbohydrates (starch), protein, and oil. Diet compositions for all trials are given in Table 1. Please note that (for purpose of clarity) our abbreviations indicate only relative differences between diets used in particular trials (*HS* ‘high starch, less protein’, *HP* ‘high protein, less starch’, *HL* ‘high lipid’), though composition of diets with the same abbreviation can vary between trials. Similarly, these abbreviations can differ from those used in previous studies on the same birds (e.g., diets HP and HS were referred to as ‘0 starch’ and ‘+ starch’ in Brzęk et al. 2009; HP diet was referred to as MS in Brzęk et al. 2010). However, for the sake of clarity, here we use the same abbreviations for diets applied in all trials. All procedures used in described trials were approved by appropriate animal care and use committees.

**Table 1:** Composition (%) of diets used in the present study and studies listed in Table 5

Species and trial/study	Diet	Proteins	Carbohydrates	Lipids
House sparrow nestlings (trials 1–2; Brzęk et al. 2009, 2011)	HP	56	0	20
	HS	44	22	8
Adult house sparrows (trial 3; Caviedes-Vidal et al. 2000)	HP	54	12	8
	HS	12	52	8
	HL	16	16	40
Nestling and adult zebra finches (trials 4–5; Brzęk et al. 2010)	HP	44	22	8
	HS	25	39	8
Chicken, quail, mallard (trials 6–8)	HP	50	12	9
	HS	10	53	8
Pine warbler (Levey et al. 1999)	Fruit	23	60	10
	Insect	53	10	32
	Seed	31	15	41
Yellow-rumped warbler (Afik et al. 1995; Ciminari et al. 2001)	Fruit	16	73	5
	Insect	53	10	32
	Seed	21	17	45
Feral pigeon (Ciminari et al. 2005)	HP	52	12	8
	HS	9	52	8

All diets used in trials 1–8 were supplemented with the same mix of vitamins, microelements, and essential amino acids (described in Lepczyk et al. 1998). Please note that some abbreviations in trials 1–2 and 4–5 differ from those used in respective papers on activity of intestinal enzymes

### Trials 1 and 2—effect of age and diet composition on pancreatic enzymes in nestling house sparrows

Pancreas samples analyzed in trials 1 and 2 came from birds that were subjects of experiments described, respectively, in Brzęk et al. (2009) and (2011). Briefly, 3-day old (in all trials, day of hatch = day 0) house sparrows were collected and hand-fed under laboratory conditions. The high-protein, starch-free diet (HP) was intended to mimic the insect-based diet typical of very young house sparrow nestlings, whereas the starch-containing diet (HS) mimicked a mixed insect-seed diet of older nestlings (however, protein content in this diet was still higher than carbohydrate content; Table 1). House sparrows in both trials were raised in almost the same way, with some differences in meal size but not in diet composition (see Brzęk et al. 2011), and showed the same diet-related changes in the mass-specific activity of intestinal maltase (Brzęk et al. 2009, 2011). Nestlings in trial 1 were analyzed at the age of 4 (sample size: diet HS 5, diet HP 4), and 12 days (5 on each diet), and in trial 2 only at the age of 12 days (sample size: diet HS 6, diet HP 5).

### Trial 3—effect of diet composition on pancreatic enzymes in adult house sparrows

Pancreas samples used in this trial came from the study described in Caviedes-Vidal et al. (2000). Briefly, 20 adult house sparrows were caught and after a 5-day long period of adjustment to laboratory conditions they were offered one of three synthetic diets, intended to mimic starchy seeds (diet HS, 7 birds), high fat seeds (diet HL, 7 birds), and insects (diet HP, 6 birds) for a period of 10 days.

### Trials 4 and 5—effect of age and diet composition on pancreatic enzymes in nestlings and adult zebra finches

Pancreas samples analyzed in these trials came from birds that were subjects of the experiments described in Brzęk et al. (2010). In trial 4, 4-day old zebra finches were collected and hand-fed under laboratory conditions. Zebra finches cannot thrive on a completely starch-free diet (Brzęk et al. 2010), and therefore they were not fed on the same diets as young house sparrows, but on those listed in Table 1. Nestlings were analyzed at the age of 5 (sample size: diet HS 9, diet HP 4), 8 (sample size: diet HS 8, diet HP 7), and 15 days (sample size: diet HS 9, diet HP 9). Comparison of 4 and 12 day old house sparrows and 5 and 15 day old zebra finches is relevant because these ages represent, respectively, 25 and 80 % of the typical fledging period in both species (Zann 1996; Anderson 2006). In trial 5, we collected pancreas samples from adult zebra finches maintained on HP or HS diets for 15–21 days (Brzęk et al. 2010). Sample size was 11 for diet HS and 9 for diet HP.

### Trials 6, 7, and 8—effect of diet composition on pancreatic enzymes in young chickens, quails and mallards

Young (1–3 day old) chickens, quails, and mallards were purchased from commercial shops and raised on commercial diet (Ganave^®^ Alimentos Pilar S.A., Argentina; composition: protein 20 %, fat 4 %, crude fiber 3.9 %). Beginning at ages 42 (chickens; trial 6), 45 (quails, trial 7) and 80 (mallards, trial 8) days, they were raised on either of two different diets (see Table 1) for 15 days (chicken and mallards) and 30 days (quail). Sample size was (HS and HP diets, respectively): 7 and 6 for chicken, 8 and 7 for quail, 7 and 7 for mallard.

### Pancreatic enzyme assays

Birds were euthanized after completion of each trial and dissected immediately. Pancreases were weighed and stored in liquid nitrogen for subsequent analyses. Samples from all trials were assayed within 2 years after being collected. Biochemical assays were carried out in Madison (USA), and San Luis (Argentina), but they followed the same procedures with minor modifications, and all assays were supervised by the same person (ECV). Below we present their general outline; more detailed description is given for trials 1–2, and 4–5 in Kohl et al. (2011), for trial 3 in Caviedes-Vidal and Karasov (2001), and for trials 6–8 in Ciminari et al. (2005). We emphasize that when we compared or combined data from different trials they were always assayed in exactly the same way (trials 1 and 2, and trials 4 and 5). In some cases, we were unable to carry out all assays for each individual (e.g., because of small size of pancreas in 5-day old zebra finches). In particular, we analyzed only amylase activity for 8-day old nestlings and adult zebra finches in trials 4 and 5.

Frozen pancreas samples were crushed into several smaller pieces. In order to avoid spatial heterogeneity in enzyme activities, 4–5 pieces from different parts of the pancreas were pooled for each enzyme assay. Activity of amylase was measured by a modification of the 3,5-dinitrosalicylate method (Dahlqvist 1962; Hjorth 1979). Pancreas samples were thawed, homogenized, and appropriately diluted aliquots were incubated with 2 % potato starch at 40 °C for 3 min. The reaction was terminated by the addition of dinitrosalicylate reagent (in blank samples, dinitrosalicylate was added before the substrate), the tubes were then incubated in boiling water for 10 min, and cooled with tap water. Finally, the absorbance was read at 530 nm. The amount of liberated glucose was estimated using glucose standard curve. Activity of pancreatic proteases was measured by a modification of (Erlanger et al. 1961) method. Pancreatic samples were homogenized, and zymogens were activated by incubation with enterokinase. The duration of zymogen activation was determined independently for each species to insure that activities of proteases were analyzed at their maximal activation (we checked which duration of zymogen activation resulted in highest activity of pancreatic proteases). Appropriately diluted aliquots were incubated with DL-BAPNA (benzoyl-arginine-*p*-nitroanilide) for trypsin or with GPNA (N-glutaryl-l-phenylalanine-*p*-nitroanilide) for chymotrypsin at 40 °C for 10 min. The reaction was terminated by adding 30 % acetic acid (in blank samples, acetic acid was added before the substrate). The liberated amount of *p*-nitroaniline was estimated by reading the absorbance at 410 nm and using a *p*-nitroaniline standard curve.

For each enzyme, we calculated its mass-specific activity (i.e., the number of moles of product formed during 1 min incubation with a mass unit of pancreatic tissue), and (in trials 1–2, 4–5) its summed activity (mass specific activity multiplied by the mass of whole pancreas). Finally, we calculated the ratios of activity of amylase to that of trypsin or chymotrypsin (hereafter referred to as A/T and A/C). These unitless ratios can be interpreted as the index of relative investments into carbohydrate- and protein-digesting enzymes. These ratios are not affected by potentially confounding factors like protein content in the analyzed sample or non-specific effects of diet on all enzymes, and thus are a good index of adaptive modulation of digestive enzymes.

### Statistics

We analyzed our data separately for each trial, either by means of two-way ANOVA (trials 1 and 4), with age and diet as main factors, or one-way ANOVA (remaining trials), with diet as the main factor. Additionally, in separate analyses we compared data for 12-day old nestlings from trials 1 and 2 by means of two-way ANOVA, with trial and diet as main factors. By conducting this analysis we tested for a diet effect in house sparrow nestlings using a larger sample size. Similarly, for mass-specific and summed activity of amylase in zebra finches, we carried out additional ANOVAs that included nestlings and adult birds (from trials 4 and 5) that were fed on the diets with the same composition. In all trials where a two-way ANOVA was applied, interactions between main factors were always non-significant (*p* > 0.05) and thus we did not include them in the final ANOVA models. If an independent variable had more than two classes (e.g., 3 diets in trial 3 or 3 ages in trial 4), statistical differences between them were identified using LSD post hoc tests. We inspected distribution of tested variables and carried out Bartlett’s and Levene’s tests to check for homogeneity of variance. We transformed our data when necessary; however, in no case did our conclusions change. Thus, for the sake of clarity, we used non-transformed data in statistical tests and figures. The standard level of significance *α* = 0.05 was applied. All analyses were carried out using SAS software.

## Results

### Trials 1 and 2—effect of age and diet composition on pancreatic enzymes in nestling house sparrows

**Fig. 1:**
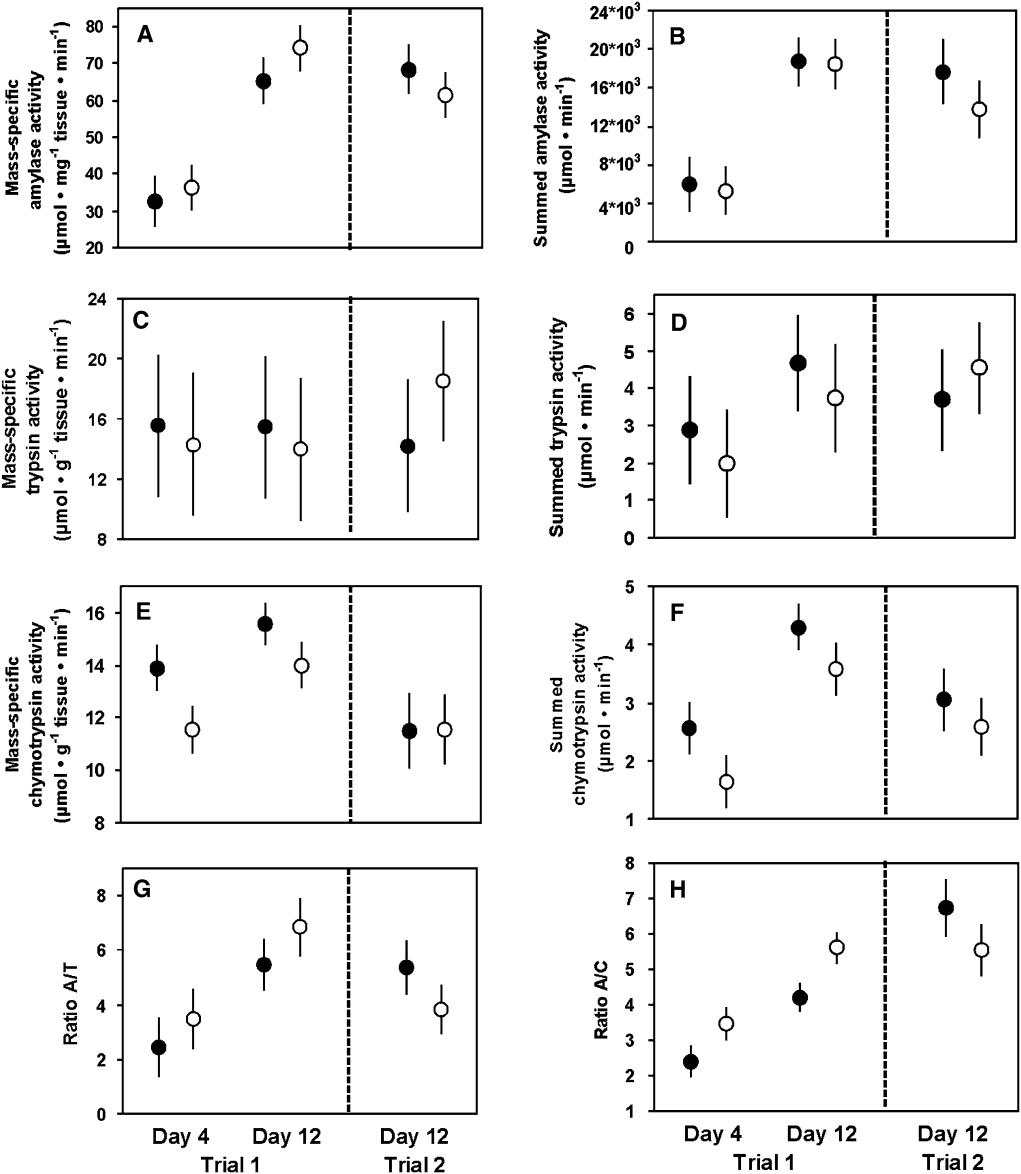
Mass-specific and summed activities of pancreatic enzymes and ratios of amylase to trypsin (A/T) and amylase to chymotrypsin (A/C) activity in nestling house sparrow in trials 1 and 2. The *filled circles* indicate birds raised on the HP diet, and the *open circles* depict birds raised on the HS diet. Mean ± S.E.M. are shown. See Tables 2 and 3 for significance of diet and age effect

Mass-specific and summed activities of amylase and chymotrypsin were significantly higher in 12-day than in 4-day old house sparrow nestlings in trial 1, although this increase was stronger in the case of amylase (Table 2; Fig. 1). On the other hand, both mass-specific and summed activities of trypsin did not change with age (Table 2; Fig. 1c, d). The highly significant increase in the activity of amylase resulted in significantly higher values of A/C and A/T ratios in older nestlings (Table 2; Fig. 1 g, h).

**Table 2:** Summary of results of ANOVAs for the effects of age and diet on activities of pancreatic enzymes in house sparrow nestlings (trial 1)

	Age		Diet	
	*F*	*p*	*F*	*p*
Mass-specific amylase activity	32.55	**<0.0001**	1.08	0.31
Summed amylase activity	26.35	**0.0001**	0.03	0.86
Mass-specific trypsin activity	0.00	0.97	0.10	0.76
Summed trypsin activity	1.76	0.21	0.47	0.50
Mass-specific chymotrypsin activity	5.95	**0.029**	5.39	**0.036**
Summed chymotrypsin activity	19.03	**0.0007**	3.71	0.075
A/T	9.94	**0.007**	1.45	0.25
A/C	21.85	**0.0004**	8.74	**0.01**

*df* for effects of age and diet: 1,16 for mass-specific and summed amylase; 1, 14 for other variables

Diet treatment did not significantly affect mass-specific or summed activities of amylase and trypsin in both trials (Tables 2,3; Fig. 1). However, nestlings raised on the HP diet in trial 1 showed higher activity of chymotrypsin than birds raised on the HS diet (this effect was significant for mass-specific activity and marginally non-significant for summed activity; Table 2; Fig. 1e, f). As a result, nestlings raised on the HP diet had significantly lower A/C but not A/T ratios (Table 2; Fig. 1g, h). However, we found no significant diet effect on activities of any enzyme or on A/C and A/T ratios in trial 2 (Table 3; Fig. 1). The effect of diet was also non-significant when data for 12-day old nestlings from both trials were analyzed by means of two-way ANOVA with trial and diet as factors (effect of diet: *p* > 0.30 for all comparisons, effect of trial was significant only for mass-specific chymotrypsin activity, interaction trial versus diet was always not significant).

**Table 3:** Summary of results of ANOVAs for the effect of diet on mass-specific and summed activities of pancreatic enzymes in house sparrow nestlings (trial 2), and mass-specific activities of pancreatic enzymes in adult house sparrows (trial 3), chickens (trial 6), quails (trial 7), and mallards (trial 8)

	Amylase		Trypsin		Chymotrypsin		A/T		A/C	
	*F*	*p*	*F*	*p*	*F*	*p*	*F*	*p*	*F*	*p*
Mass-specific activity in nestling house sparrows	0.60	0.46	0.57	0.47	0.00	0.98	1.89	0.20	0.78	0.40
Summed activity in nestling house sparrows	0.74	0.41	0.14	0.72	0.23	0.64				
Adult house sparrows	5.95	**0.011**	2.44	0.12	0.84	0.45	2.09	0.16	5.11	**0.018**
Chicken	1.10	0.32	0.00	0.99	0.60	0.46	1.31	0.28	0.49	0.50
Quail	0.23	0.64	1.75	0.21	0.70	0.42	2.09	0.17	0.01	0.91
Mallard	1.72	0.21	2.55	0.14	8.98	**0.011**	4.33	0.06	6.58	**0.025**

*df*: 1, 9 for nestling sparrows; 2, 17 for adult sparrows (2, 16 for trypsin and A/T); 1, 11 for chicken; 1, 13 for quail; 1, 12 for mallard

### Trial 3—effect of diet composition on pancreatic enzymes in adult house sparrow

**Fig. 2:**
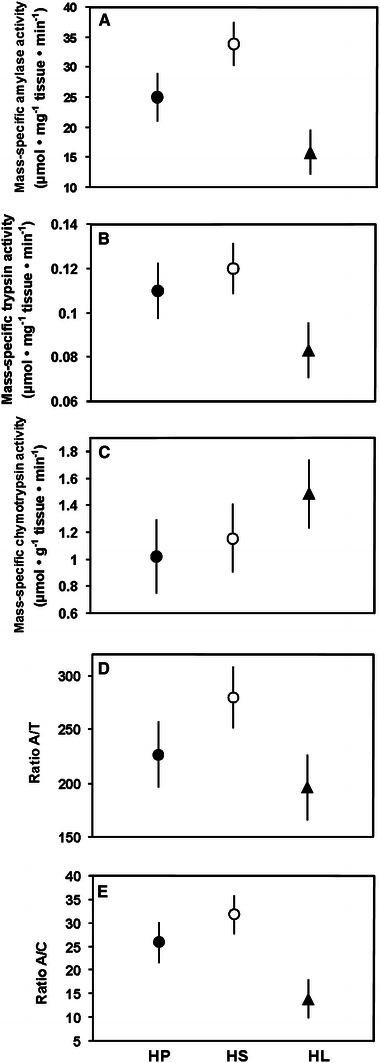
Mass-specific activities of pancreatic enzymes and ratios of amylase to trypsin (A/T) and amylase to chymotrypsin (A/C) activity in adult house sparrow in trial 3. The *filled circles* indicate birds fed on the HP diet, the *open circles* depict birds fed on the HS diet, and the *filled triangles* indicate birds fed on the HL diet. Mean ± S.E.M. are shown. See Table 3 for significance of diet effect

Dietary treatment significantly affected mass-specific activity of amylase in adult house sparrows (Table 3; Fig. 2a). Activity of amylase ranked in the order HS > HP > HL, but only the difference between HS and HL groups was significant (*p* = 0.0031), whereas the HP diet did not differ from other diets (0.11 < *p* < 0.12 for both comparisons). Mass-specific activities of trypsin and chymotrypsin did not depend on diet manipulation (Table 3; Fig. 2b, c). Diet composition affected values of A/C but not A/T ratios (Table 3; Fig. 2d, e). Sparrows fed on the HL diet had lower A/C ratio than those fed on the HS diet (*p* = 0.0059), and marginally non-significantly lower than the HP group (*p* = 0.058), whereas there was no difference between birds fed on HS and HP diets (*p* = 0.34).

### Trials 4 and 5—effect of age and diet composition on pancreatic enzymes in adult and nestling zebra finch

**Fig. 3:**
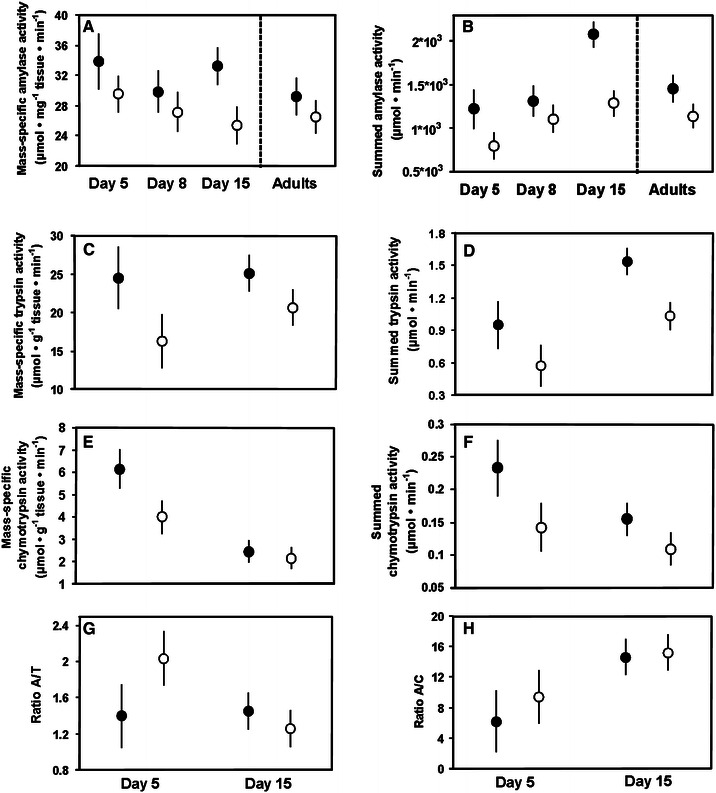
Mass-specific and summed activities of pancreatic enzymes and ratios of amylase to trypsin (A/T) and amylase to chymotrypsin (A/C) activity in nestling and adult zebra finches in trials 4 and 5. The *filled circles* indicate birds raised on the HP diet, and the *open circles* depict birds raised on the HS diet. Mean ± S.E.M. are shown. See Table 4 and text for significance of diet and age effect

Mass-specific activities of pancreatic enzymes either did not change (amylase and trypsin) or decreased (chymotrypsin) between 5 and 15 days of age in young zebra finches in trial 4 (Table 4; Fig. 3a, c, e). A simultaneous increase in pancreas size resulted in higher summed activities of amylase and trypsin in 15-day old nestlings (for amylase, *p* < 0.01 for comparison of day 15 with both other ages, *p* = 0.28 for comparison of day 5 with day 8), whereas summed activity of chymotrypsin did not change with age (Table 4; Fig. 3b, d, e). A significant decrease in the mass-specific activity of chymotrypsin with age was responsible for a simultaneous increase in the A/C ratio, whereas the A/T ratio did not change with age (Table 4; Fig. 3g, h). The HP diet usually significantly or near-significantly increased both mass-specific and summed activities of all pancreatic enzymes in zebra finch nestlings, with chymotrypsin being least affected (Table 4; Fig. 3). However, A/T and A/C ratios were not modulated by diet composition (Table 4; Fig. 3g, h).

**Table 4:** Summary of results of ANOVAs for the effects of age and diet on activities of pancreatic enzymes in zebra finch nestlings (trial 4)

	Age		Diet	
	*F*	*p*	*F*	*p*
Mass-specific amylase activity	0.63	0.54	4.78	**0.034**
Summed amylase activity	7.90	**0.0012**	12.30	**0.0011**
Mass-specific trypsin activity	0.76	0.39	3.93	0.06
Summed trypsin activity	9.75	**0.0050**	9.98	**0.0046**
Mass-specific chymotrypsin activity	15.61	**0.0007**	1.74	0.20
Summed chymotrypsin activity	2.61	0.12	4.00	0.058
A/T	2.10	0.16	0.02	0.88
A/C	5.29	**0.031**	0.23	0.63

*df*: for age 2, 42 (mass-specific and summed amylase), or 1, 22 (other variables); for diet 1, 42 (mass-specific and summed amylase), or 1, 22 (other variables)

Diet treatment did not affect mass-specific or summed activities of amylase in adult zebra finches in trial 5 (*F*_1, 18_ = 1.14, *p* = 0.30, and *F*_1, 18_ = 2.49, *p* = 0.13, respectively; Fig. 3a, b). When data for all nestling and adult zebra finches were analyzed together, age still had no significant effect on mass-specific activity of amylase (*F*_3, 61_ = 0.75, *p* = 0.52; Fig. 3a), whereas the effect of age on summed activity of amylase remained significant (*F*_3, 61_ = 5.73, *p* = 0.0016), reflecting higher pancreas masses in 15-day old nestlings than in birds of all other ages (*p* < 0.015 for comparison of day 15 with all other ages, *p* > 0.05 for all comparisons between remaining age classes; Fig. 3b). However, both mass-specific (*F*_1, 61_ = 5.95, *p* = 0.018), and summed (*F*_1, 61_ = 14.60, *p* = 0.0003) activities of amylase were higher in birds fed on the HP diet (Fig. 3a, b).

### Trials 6, 7, 8—effect of diet composition on pancreatic enzymes in young chickens, quails, and mallards

**Fig. 4:**
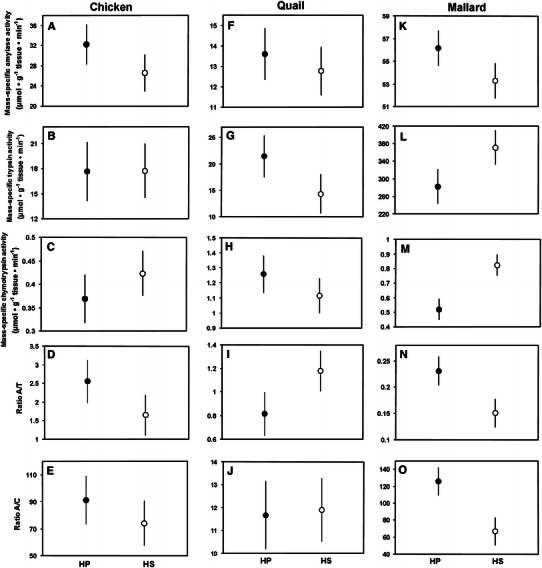
Mass-specific activities of pancreatic enzymes and ratios of amylase to trypsin (A/T) and amylase to chymotrypsin (A/C) activity in chickens (*left column*; trial 6), quails (*central column*; trial 7), and mallards (*right column*; trial 8). The *filled circles* indicate birds fed on the HP diet, and the *open circles* depict birds fed on the HS diet. Mean ± S.E.M. are shown. See Table 3 for significance of diet effect

Diet had no significant effect on mass-specific enzyme activities, nor on A/C and A/T ratios in chickens and quails (Table 3; Fig. 4). In mallards, birds fed on the HP diet showed lower activity of chymotrypsin, and significantly or almost-significantly higher values of A/T and A/C ratios (Table 3; Fig. 4m, n, o). The HP diet also significantly increased values of A/C in chickens and A/T in mallards when one outlying point was excluded for each species. However, in all cases the direction of these diet-induced changes was opposite to that predicted by the adaptive modulation hypothesis.

## Discussion

### Effect of age on pancreatic enzymes in house sparrow and zebra finch nestlings

In trial 1, we found a highly significant increase in mass-specific activity of amylase, as well as values of A/C and A/T ratios in house sparrow nestlings between 4 and 12 days of life (Fig. 1). The same pattern was observed in an earlier study of this species (Caviedes-Vidal and Karasov 2001). On the other hand, age had no significant effect on the mass-specific activity of amylase in zebra finch nestlings in trial 4; remarkably, mass-specific activity of amylase in young, 5-day old nestlings did not differ from that observed in adult birds in trial 5 (Fig. 3a). We hypothesize that this lack of an age effect reflects the high specialization for digestion of carbohydrate-rich food in the zebra finch, with some crucial enzymes already reaching their functional maturity in very young nestlings. Similarly, the increase in mass-specific activity of intestinal maltase during the nestling period is relatively greater in the house sparrow (Caviedes-Vidal and Karasov 2001; Brzęk et al. 2009), than in the zebra finch (Brzęk et al. 2010). We conclude that ontogenetic changes in mass-specific activity of amylase in both species are matched to changes in their typical dietary starch content and roughly parallel changes in the activity of intestinal maltase (Brzęk et al. 2009, 2010).

Age had no consistent effect on mass-specific activities of pancreatic proteases in young house sparrows and zebra finches (Figs. 1,3), despite weak but significant increases in the mass-specific activity of intestinal APN in both species (Brzęk et al. 2009, 2010). Thus, ontogenetic changes in activities of pancreatic proteases were less correlated to simultaneous changes in intestinal proteases than in the case of carbohydrases; they also matched nestlings’ diet composition less closely. Interestingly, mass-specific activity of pancreatic and intestinal proteases seem to be uncorrelated on an inter-specific level in adult passerines (see Fig. 3 in Kohl et al. 2011). Similarly, in many groups of animals the activities of proteases are less correlated to diet composition than are activities of carbohydrases (for examples and discussion of possible explanations see Karasov et al. 2011; Kohl et al. 2011). However, the mass of pancreatic tissue increased with age in both house sparrow and zebra finch nestlings (Brzęk et al. 2009, 2010). As a result, the summed activity of trypsin (but not chymotrypsin) was higher in older zebra finch nestlings (Fig. 3d), and thus better matched a simultaneous increase in summed activity of intestinal APN (Brzęk et al. 2010). Similarly, summed amylase activity (which presumably better quantifies digestive capacity than mass-specific activity) increased with age in this species (Fig. 3b), which could improve the capacity for starch digestion in older nestlings.

### Effect of diet composition on pancreatic enzymes

We found little evidence for the presence of adaptive, diet-induced modulation of pancreatic enzymes in the five studied species (Tables 2, 3, 4, Figs. 1, 2, 3, 4). The only exception was the elevated activity of chymotrypsin and reduced value of A/C ratio in house sparrow nestlings fed on a diet with higher dietary protein content in trial 1; however, this result was not repeated in trial 2 (Tables 2,3; Fig. 1e, h). In all other cases, mass-specific activities of pancreatic enzymes were either not affected by diet composition, changed in direction opposite to that predicted by adaptive modulation hypothesis, or responded non-specifically to diet manipulation. The last response is best exemplified by nestling zebra finches, in that the HP diet elevated mass-specific amylase activity and tended to increase mass-specific trypsin activity (Table 4; Fig. 3a, c). As a result, A/T ratio was not affected and activity of amylase was higher in birds fed on diet with lower carbohydrate content, clearly suggesting a non-adaptive response to diet manipulation.

The power of statistical analyses in our trials was limited by small sample size. We estimated that, given the intra-group variation observed in 12-day old house sparrow nestlings in trials 1 and 2, we could not detect a relative difference between diets in mass-specific amylase activity smaller than 34 % in a single trial and 21 % when data from both these trials were pooled together. However, the same 12-day old house sparrow nestlings fed on the HS diet showed at least twofold increase in mass-specific activity of intestinal maltase when compared to those fed on the HP diet (Brzęk et al. 2009, 2011). Thus, the effect of diet composition on mass-specific activity of amylase in young house sparrows is considerably weaker than in the case of intestinal maltase. Moreover, sample sizes in several trials were large enough to detect significant changes in pancreatic enzymes in direction opposite to that predicted by adaptive modulation hypotheses or changes in activities of intestinal enzymes (see Table 5).

**Table 5:** Summary of studies testing the effect of diet composition on mass-specific activities of pancreatic and intestinal enzymes

Species	Effect of dietary carbohydrates		Effect of dietary proteins	
	Pancreatic amylase	Intestinal maltase	Pancreatic proteases	Intestinal APN
Nestling sparrows	ns	↑	Chymotrypsin (↑)	ns
Adult sparrows	ns	ns^a^	ns	↑
Zebra finch nestlings	↓	ns	Trypsin (↑)	ns
Zebra finch adults	ns	↓	Not tested	↑
Chicken	ns	↑	ns	ns
Quail	ns	↑	ns	ns
Mallard	ns	↑	Chymotrypsin ↓	↑
Pine warbler	↑^b^	↑^c^	↑	↑
Yellow-rumped warbler	ns	ns	Trypsin (↓)	↑
Feral pigeon	ns	ns	Chymotrypsin (↓)	↑

*Upward arrows* indicate significant increase in agreement with adaptive modulation hypothesis, *downward arrows* indicate significant decrease opposite to adaptive modulation hypothesis, *arrows* in *brackets* indicate not repeatable pattern or a near-significant trend (0.05 < *p* < 0.1) in relevant direction

Data from the present study and from Brzęk et al. 2009, 2011 (intestinal enzymes in house sparrow nestlings); Caviedes-Vidal et al. 2000 (intestinal enzymes in adult house sparrows); Brzęk et al. 2010 (intestinal enzymes in nestling and adult zebra finches); Ciminari unpublished PhD thesis (intestinal enzymes in chicken, quail, mallard); Levey et al. 1999 (all enzymes in pine warbler); Ciminari et al. 2001 (pancreatic enzymes in yellow-rumped warbler); Afik et al. 1995 (intestinal enzymes in yellow-rumped warbler); Ciminari et al. 2005 (all enzymes in feral pigeon)

^a^Significant effect of lipids on amylase and maltase activity

^b^Low carbohydrate content did not always decrease activity of amylase

^c^Seed diet reduced activity of maltase but not amylase when compared to fruit diet, possible effect of varying lipid level between diets

Interestingly, high dietary lipid content significantly reduced activity of amylase in adult house sparrows in trial 3 (Table 3; Fig. 2a). In the same birds, the HL diet significantly reduced activity of intestinal maltase (Caviedes-Vidal et al. 2000). Such dietary lipid-induced decrease in activities of carbohydrases has been observed in many other studies, although its mechanism and potential adaptive value are unknown (Karasov et al. 2011). However, this effect may suggest the presence of regulatory pathways that can modulate activity of pancreatic enzymes in altricial birds in response to nutrients other than their specific substrates.

The lack of adaptive, diet-mediated flexibility of any studied pancreatic enzyme in three galloanserine species in trials 6–8 (Table 3; Fig. 4) is somewhat puzzling because several studies have shown that activity and/or secretion rate of proteases (Imondi and Bird 1967; Dunnington and Siegel 1995), and lipase (Hulan and Bird 1972; Dror et al. 1976; Dunnington and Siegel 1995; Maiorka et al. 2004) can be adaptively modulated by diet composition in galliforms. Similarly, diet quantity (Hulan and Bird 1972), quality (Jang et al. 2004; Lin et al. 2010), or the presence of external amylase in consumed food (Jiang et al. 2008) can affect rates of synthesis or secretion of amylase in poultry. However, none of these earlier studies directly tested the effect of varying protein/carbohydrate ratio on activity of pancreatic amylase. Moreover, most of these studies focused on only one enzyme, and thus they cannot exclude the possibility of non-specific diet effects (e.g., higher dietary protein content increases activity of both pancreatic proteases and carbohydrases in Peking duck; Zhao et al. 2007). Thus, we conclude that there is no unequivocal evidence that dietary carbohydrate/protein ratio adaptively modulates pancreatic amylase in galloanserine birds, although activity of other pancreatic enzymes can be adaptively modulated by other dietary characteristics.

### Why does diet composition have little adaptive effect on the activity of pancreatic enzymes in birds?

Diet-induced, adaptive changes in activities of pancreatic enzymes have been observed in many groups of animals (mammals, birds, reptiles, fish, insects, crustaceans; reviewed by Brannon 1990; Karasov and Hume 1997; Karasov et al. 2011). However, we found little evidence for their presence in our study. For example, nestling house sparrows possess higher flexibility of intestinal maltase than adult birds (Caviedes-Vidal et al. 2000; Brzęk et al. 2009, 2011); but neither age class showed adaptive plasticity of amylase. Thus, young house sparrows fed on the HP diet either synthesised amylase far in excess of their needs or (less likely) nestlings fed on the HS diet synthesised less amylase than they needed. Both situations contradict predictions of ‘adaptive modulation’ hypothesis.

In Table 5, we summarize findings of the present paper and similar experiments that analyzed the effect of carbohydrate/protein/lipid content in diet on the activity of pancreatic enzymes in birds, and compared them with simultaneous changes in activity of intestinal enzymes. We found almost no unambiguous example of diet-mediated, adaptive modulation of pancreatic carbohydrases and proteases. In the case of nestling zebra finches, activities of amylase and trypsin changed in the same directions and A/T ratio was not affected. In the yellow-rumped warbler, activity of trypsin was elevated on a seed diet that was not richest in protein (Ciminari et al. 2001), and in the pine warbler the seed diet was poor in carbohydrates but did not reduce amylase activity when compared with a fruit diet (Levey et al. 1999; see Table 1). Although sometimes the lack of a diet effect on enzyme activity can be predicted (e.g., high-carbohydrate fruits used in experiments with both warbler species contained presumably mostly mono- and di-saccharides and thus, amylase was not necessary for their digestion), this review shows that adaptive modulation of pancreatic enzymes in birds seems to be surprisingly rare. The only case when both pancreatic and intestinal enzymes changed adaptively was found for proteases in pine warbler.

An important caveat is that all studies in Table 5 analyzed activity of enzymes in pancreatic tissue, whereas functional activity of these enzymes depends on the rate of their secretion into the intestinal lumen and (for proteases) on the rate of their activation, all in relation to rates of digesta inflow from the stomach. Indeed, diet- or age-related changes in enzyme activity can be different in pancreatic tissue and in intestinal lumen (Nir et al. 1993; Kadhim et al. 2011). Mechanisms controlling the secretion of pancreatic enzymes in birds involve both the endocrine and nervous systems (Wang and Cui 2007), and presumably enable fine adjustment of enzyme concentration in the intestinal lumen. We cannot exclude the possibility that diet manipulation affected only the rate of enzyme secretion; however, several studies listed in Table 5 found that diet composition affected activities of enzymes in pancreatic tissue, although usually in a non-adaptive way. Moreover, there is a strong, inter-specific correlation between activity of amylase in pancreatic tissue and diet carbohydrate content in adult passerines (Kohl et al. 2011).

Perhaps the most interesting conclusion of the review presented in Table 5 is that changes in activities of pancreatic and intestinal enzymes processing the same dietary component are usually not correlated. Paradoxically, a perfect match between changes in pancreatic and intestinal carbohydrases in adult house sparrows fed on the HL diet presumably reflects a non-adaptive response to diet composition. Similarly, there is no correlation between seasonal changes in activities of amylase and maltase in wild Western sandpipers (Stein et al. 2005). These results suggest that activities of pancreatic and intestinal digestive enzymes in birds are controlled and modulated independently. This is perhaps not an unexpected conclusion because, as we explained earlier, the functional activity of intestinal enzymes reflects mainly the rate of their synthesis in intestinal epithelium, whereas functional activity of pancreatic enzymes may depend both on the rate of their synthesis and on the rate of their secretion into the intestinal lumen and/or activation. However, our earlier experiments revealed the presence of an internal, genetic program responsible for ontogenetic increase in the activity of maltase in house sparrow, which is significantly modified by diet composition (Brzęk et al. 2009, 2011). In contrast, results of trials 1–2 in the present study suggest that the simultaneous increase in amylase activity is exclusively under genetic control. This is an unexpected conclusion, because both enzymes cooperate closely during food hydrolysis.

We cannot offer an unambiguous explanation for the presumably low adaptive flexibility of pancreatic enzymes in birds. The functional activity of pancreas has been much less studied in birds than in mammals, yet results suggest that it differs considerably between the two taxa (Wang and Cui 2007). The human pancreas is said to possess an enormous, tenfold excess capacity (DiMagno et al. 1973), which represents one of the highest safety factors observed in any biological structure (see Fig. 26 in Piersma and van Gils 2010). If similar excess capacity occurs in the avian pancreas, there may be little selection pressure for diet-induced enzyme modulation (although it is not immediately clear why natural selection allows for such an apparent waste of resources). However, the presence of inter-specific correlation between dietary starch content and activity of amylase in adult passerines (Kohl et al. 2011), strongly suggests that birds are more prudent in their resource management. Similarly, different ontogenetic patterns of changes in amylase activity in house sparrow and zebra finches can be best explained by matching the rate of amylase synthesis to starch content in diet. Finally, the addition of external amylase to the diet can improve body mass increments in chickens even though synthesis of pancreatic amylase is down-regulated (Jiang et al. 2008), suggesting that cost of amylase production is not trivial.

In summary, we showed that ontogenetic profiles of mass-specific activity of amylase in house sparrow and zebra finch match starch content in typical nestlings’ diet, as well as simultaneous changes in activity of intestinal carbohydrases. However, most of our trials showed no evidence for the presence of diet-related, adaptive modulation of activity of pancreatic enzymes. Moreover, changes in mass-specific activity of pancreatic enzymes were usually nor correlated with simultaneous changes in activity of intestinal enzymes. The most plausible explanation is that the mass-specific activity of pancreatic enzymes in birds is controlled almost exclusively by intrinsic genetic mechanisms. Therefore, although it may be matched evolutionary to typical diet composition, it is less capable for diet-related flexibility. However, more work is needed to investigate the generality of this conclusion.
